# Recreational Marijuana Use and Acute Myocardial Infarction: Insights from Nationwide Inpatient Sample in the United States

**DOI:** 10.7759/cureus.1816

**Published:** 2017-11-03

**Authors:** Rupak Desai, Upenkumar Patel, Shobhit Sharma, Parth Amin, Rushikkumar Bhuva, Malav S Patel, Nitin Sharma, Manan Shah, Smit Patel, Sejal Savani, Neha Batra, Gautam Kumar

**Affiliations:** 1 Research Coordinator, Atlanta Veterans Affairs Medical Center; 2 Public Health, National University; 3 Biology, Texas State University; 4 Department of Internal Medicine, Wellstar Atlanta Medical Center; 5 Internal Medicine, Canton Medical Education Foundation; 6 Department of Healthcare Administration and Health Informatics, Marshall University; 7 Internal Medicine, University of Texas, Houston; 8 Department of Neurology, University of Connecticut Health Center; 9 New York University; 10 Pediatrics, St. Joseph Regional Medical Center; 11 Department of Medicine, Division of Cardiology, Atlanta Veterans Affairs Medical Center and Emory University School of Medicine

**Keywords:** marijuana, cannabis, acute myocardial infarction, substance abuse, mortality, trend, myocardial infarction, nationwide, cardiovascular, recreational

## Abstract

Background

Marijuana is a widely used recreational substance. Few cases have been reported of acute myocardial infarction following marijuana use. To our knowledge, this is the first ever study analyzing the lifetime odds of acute myocardial infarction (AMI) with marijuana use and the outcomes in AMI patients with versus without marijuana use.

Methods

We queried the 2010-2014 National Inpatient Sample (NIS) database for 11-70-year-old AMI patients. Pearson Chi-square test for categorical variables and Student T-test for continuous variables were used to compare the baseline demographic and hospital characteristics between two groups (without vs. with marijuana) of AMI patients. The univariate and multivariate analyses were used to assess and compare the clinical outcomes between two groups. We used Cochran–Armitage test to measure the trends. All statistical analyses were executed by IBM SPSS Statistics 22.0 (IBM Corp., Armonk, NY). We used weighted data to produce national estimates in our study.

Results

Out of 2,451,933 weighted hospitalized AMI patients, 35,771 patients with a history of marijuana and 2,416,162 patients without a history of marijuana use were identified. The AMI-marijuana group consisted more of younger, male, African American patients. The length of stay and mortality rate were lower in the AMI-marijuana group with more patients being discharged against medical advice. Multivariable analysis showed that marijuana use was a significant risk factor for AMI development when adjusted for age, sex, race (adjusted OR 1.079, 95% CI 1.065-1.093, p<0.001); adjusted for age, female, race, smoking, cocaine abuse (adjusted OR 1.041, 95% CI 1.027-1.054, p<0.001); and also when adjusted for age, female, race, payer status, smoking, cocaine abuse, amphetamine abuse and alcohol abuse (adjusted OR: 1.031, 95% CI: 1.018-1.045, p<0.001). Complications such as respiratory failure (OR 18.9, CI 15.6-23.0, p<0.001), cerebrovascular disease (OR 9.0, CI 7.0-11.7, p<0.001), cardiogenic shock (OR 6.0, CI 4.9-7.4, p<0.001), septicemia (OR 1.8, CI 1.5–2.2, p<0.001), and dysrhythmia (OR 1.8, CI 1.5-2.1, p<0.001) were independent predictors of mortality in AMI-marijuana group.

Conclusion

The lifetime AMI odds were increased in recreational marijuana users. Overall odds of mortality were not increased significantly in AMI-marijuana group. However, marijuana users showed higher trends of AMI prevalence and related mortality from 2010-2014. It is crucial to assess cardiovascular effects related to marijuana overuse and educate patients for the same.

## Introduction

In addition to alcohol and tobacco, cannabis is one of the most abused substances globally [1]. It is an extensively used recreational substance [2]. Few states in the United States of America (USA) currently have laws broadly legalizing marijuana in some form. With the rise in cannabis legalization across the USA, the prevalence of marijuana use is expected to grow. Some studies reported that marijuana use affects the cardiovascular system, posing a risk in older people with coronary heart disease and with tachycardia at rest [3]. In other study and few case reports, the risk of myocardial infarction increased nearly five times after 1-hour post marijuana smoking when compared to marijuana non-smoking period [4, 5]. The results of long-term use of marijuana have been reported in very few studies, with no increased risk of mortality in the age group of younger than 50 years [6]. However, the literature remains contentious with regard to this issue and additional studies are required to elucidate the potential relationship between marijuana use and cardiovascular events. The current study was designed to analyze the acute myocardial infarction (AMI) prevalence with marijuana use, the odds of AMI incidence with marijuana use, and the predictors of inpatient mortality in AMI with marijuana use.

## Materials and methods

We analyzed a population-based sample of AMI patients from the 2010 to 2014 National Inpatient Sample (NIS) Database of the Healthcare Cost and Utilization Project (HCUP) sponsored by Healthcare Research and Quality Agency (AHRQ). NIS is the largest all-payer inpatient healthcare database which is publicly accessible in the United States. NIS database comprises a 20% sample inpatient from 1000 hospitals of more than 40 states which contain an average of seven million unweighted discharges per year. This data estimate results for more than 35 million weighted hospitalizations of the US population [7].

In our study, the Clinical Classifications Software (CCS) diagnosis code 100 was used to recognize the AMI patients aged 11 to 70 years. Of these, histories of marijuana dependence and non-dependent marijuana use were identified using International Classification of Diseases, Ninth Revision, Clinical Modification (ICD-9-CM) codes 304.30, 304.31, 304.32, 305.20, 305.21, and 305.22. The approach has already been used before to precisely distinguish patients with a history of recreational use of marijuana [8]. Moreover, we examined patients’ baseline demographic and hospital details between two groups AMI-marijuana vs. AMI-non marijuana as shown in Table 1.

**Table 1:** Baseline Characteristics of Hospitalized Acute Myocardial Infarction Patients with known Marijuana Use SNF: skilled nursing facility, ICF: intermediate care facility

Variables	AMI without Marijuana	AMI with Marijuana	P-value*
Unweighted Index admissions	487,317	7,202	
Weighted Index admissions	2,416,162	35,771	
Age in years at admission			<0.001
Mean age ± SD	57.79 ± 8.98	49.34 ± 10.80	
11-25	0.3%	2.5%	
26-40	4.2%	18.0%	
41-55	31.0%	50.1%	
56-70	64.5%	29.4%	
Weekend Admissions			<0.001
Monday-Friday	74.2%	71.6%	
Saturday-Sunday	25.8%	28.4%	
Died during hospitalization			<0.001
Did not die	94.2%	96.6%	
Died	5.8%	3.4%	
Disposition of Patient			<0.001
Routine	66.0%	74.0%	
Transfer to short-term hospital	8.6%	6.9%	
Other Transfers (SNF, ICF, another facility)	9.1%	4.9%	
Home Health Care	9.3%	6.8%	
Against Medical Advice (AMA)	1.2%	4.0%	
Died	5.8%	3.4%	
Elective vs. Non-elective Admissions			<0.001
Non-elective	91.7%	94.8%	
Elective	8.3%	5.2%	
Indicator of Sex			<0.001
Male	66.0%	76.9%	
Female	34.0%	23.1%	
Length of stay (cleaned)			<0.001
Mean length of stay (days) ±SD	5.65 ± 8.01	4.74 ± 5.94	
≤3 days	54.4%	58.7%	
4 to 6 days	20.3%	21.8%	
7 to 9 days	10.1%	8.8%	
10 to 12 days	5.5%	4.5%	
≥13 days	9.7%	6.2%	
Primary Expected Payer			<0.001
Medicare	36.6%	20.6%	
Medicaid	11.9%	26.9%	
Private including HMO	37.4%	22.5%	
Self - Pay	9.3%	22.9%	
No charge	0.9%	2.2%	
Other	3.9%	4.9%	
Race			<0.001
White	71.4%	55.4%	
Black	13.8%	33.0%	
Hispanic	8.4%	6.8%	
Asian or Pacific Islander	2.4%	0.7%	
Native American	0.7%	0.9%	
Other	3.4%	3.3%	
Median Household Income Quartile on Patient’s ZIP			<0.001
$1 - $39, 999	32.2%	44.0%	
$40, 000 - $50,999	27.0%	26.4%	
$51, 000 - $65, 999	23.2%	18.9%	
$66, 000 +	17.7%	10.6%	
Total charges (Mean)	$85,702.22	$76,272.23	<0.001
Bed Size of Hospital^¥ ^			0.001
Small	10.6%	10.0%	
Medium	24.5%	24.4%	
Large	64.9%	65.6%	
Location/Teaching Status of Hospital			<0.001
Rural	8.9%	6.3%	
Urban - non teaching	36.7%	32.1%	
Urban - teaching	54.4%	61.7%	
*Significant P-values ≤ 0.05 at 95% confidence Interval, ¥ The bed size cutoff points divided into small, medium, and large have been done so that approximately one-third of the hospitals in a given region, location, and teaching status combination would fall within each bed size category. Derived from https://www.hcupus.ahrq.gov/db/vars/hosp_bedsize/nisnote.jsp			

AHRQ co-morbidities and other related co-morbidities were also scrutinized as shown in Table 2.

**Table 2:** Comorbidities in Hospitalized Acute Myocardial Infarction Patients with known Marijuana Use

Variables	AMI without Marijuana	AMI with Marijuana	P-value*
Comorbidities^#^			
AIDS	0.3%	1.0%	<0.001
Motor Vehicle Accident	0.2%	0.2%	0.218
Septicemia	8.2%	5.0%	<0.001
Musculoskeletal			
RA/CVD	2.3%	1.5%	<0.001
Cardiovascular			
Congestive Heart Failure	7.6%	5.8%	<0.001
Dyslipidemia	58.9%	50.6%	<0.001
Atherosclerosis	8.7%	6.6%	<0.001
Cardiomyopathy	8.4%	12.1%	<0.001
Thromboembolism	4.3%	3.5%	<0.001
Previous MI	10.7%	12.9%	<0.001
Family History of CAD	10.7%	13.7%	<0001
Previous PCI	15.6%	14.5%	<0.001
Previous CABG	6.7%	3.8%	<0.001
Cardiogenic Shock	5.4%	3.5%	<0.001
History of SCA	0.6%	0.7%	<0.001
Valvular Disease	1.9%	1.6%	<0.001
Peripheral Vascular Disorders	10.6%	7.9%	<0.001
Hypertension	67.6%	62.8%	<0.001
Dysrhythmia	25.6%	23.0%	<0.001
Vasopressor Infusion	1.4%	1.1%	<0.001
Post-MI Dressler’s Syndrome	0.3%	0.4%	<0.001
Left Ventricular Failure	0.1%	0.1%	0.628
Respiratory			
Chronic Pulmonary Disease	21.5%	22.4%	<0.001
Pulmonary Circulation Disorders	1.7%	1.0%	<0.001
Respiratory Failure	18.7%	15.8%	<0.001
Pneumonia	9.3%	7.0%	<0.001
Obstructive Sleep Apnea	8.1%	5.2%	<0.001
Neurological			
Paralysis	2.3%	1.6%	<0.001
Other Neurological Disorders	5.4%	6.5%	<0.001
Cerebrovascular Disease	2.8%	2.7%	0.133
Transient Ischemic Attacks	0.3%	0.2%	0.057
Psychiatry			
Depression	8.6%	10.9%	<0.001
Psychoses	3.5%	8.4%	<0.001
Alcohol Abuse	5.1%	22.6%	<0.001
Smoking	46.3%	75.9%	<0.001
Cocaine Abuse	1.2%	18.9%	<0.001
Amphetamine Abuse	0.3%	5.6%	<0.001
Drug Abuse	3.3%	99.5%	<0.001
Hemato-oncological			
Deficiency Anemia	15.7%	12.1%	<0.001
Coagulopathy	6.6%	6.1%	<0.001
Weight Loss	4.2%	3.6%	<0.001
Metastatic Cancer	1.2%	0.4%	<0.001
Solid Tumor without Metastasis	1.3%	0.7%	<0.001
Endocrinological			
Diabetes, Uncomplicated	30.0%	18.3%	<0.001
Diabetes with Chronic Complications	8.4%	4.7%	<0.001
Hypothyroidism	7.8%	3.7%	<0.001
Renal			
Renal Failure	16.5%	10.3%	<0.001
Fluid and Electrolyte Disorders	25.4%	27.3%	<0.001
Hemodialysis	5.4%	2.7%	<0.001
Gastrointestinal			
Liver Disease	2.7%	4.5%	<0.001
Obesity	19.2%	15.3%	<0.001
*Significant P-values ≤ 0.05 at 95% confidence Interval, ^#^Variables are AHRQ Co-morbidity measures			
Abbreviations: AIDS – Acquired Immunodeficiency Syndrome, RA/CVD – Rheumatoid Arthritis/ Collagen Vascular Disease, MI – Myocardial Infarction, CAD – Coronary Artery Disease, PCI – Percutaneous Coronary Intervention, CABG – Coronary Artery Bypass Grafting, SCA – Sudden Cardiac Arrest			

**Figure 1:**
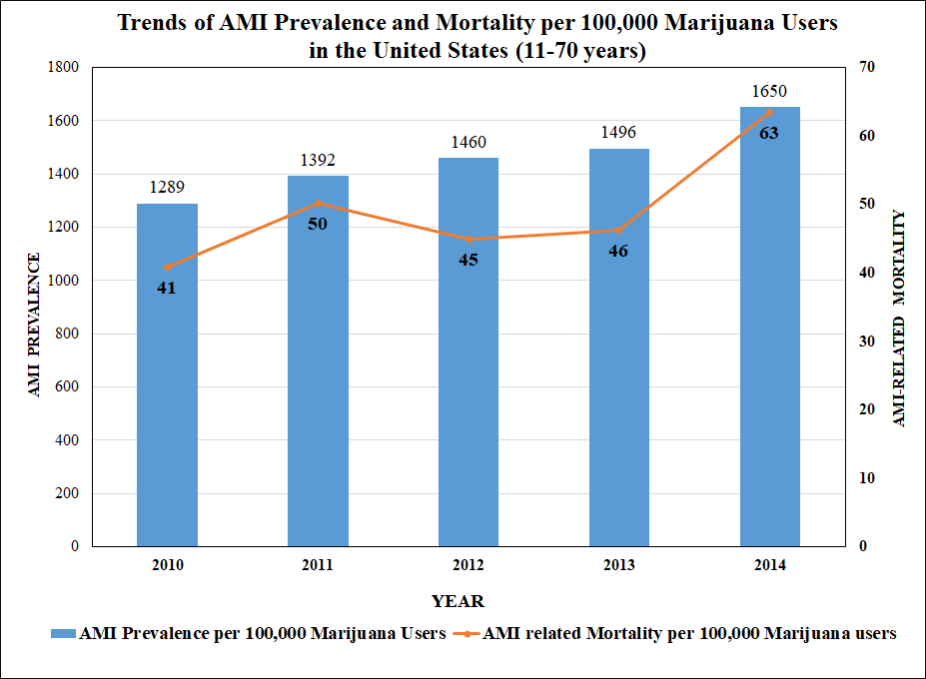
Trends of AMI Prevalence and Mortality per 100,000 Marijuana Users in the United States

We also assessed the trends of AMI prevalence and AMI-related mortality in 11-70 years old marijuana users as shown in Figure 1.

The primary outcome in our study was the prevalence of AMI, predictors of AMI incidence and inpatient mortality and secondary outcomes included the length of stay (LOS), total hospital charges, and complications. We used ICD-9 CM codes and Clinical Classification Software codes to identify the comorbidities which were not classified in NIS database shown in the appendix.

*Pearson chi-square test* for categorical variables and *Student t-test* for continuous variables were used to compare the baseline demographic and hospital characteristics between two groups (without vs. with marijuana) of AMI patients. A two side tailed p-value <0.05 was considered to determine the statistical significance. The categorical variables were measured in percentages, and continuous variables were expressed in mean±SD. We evaluated odds of AMI incidence and in-hospital mortality in AMI with marijuana use by univariate analysis and then clinically significant relevant patients’ variables were incorporated in multivariate logistic regression after adjusting for potential confounders including demographic and hospital criteria, history of substance abuse, and other relevant comorbidities. Odds ratio, 95% confidence interval, and p-value were used to report logistic regression results. The multivariate regression model was adjusted for potential confounders such as age, race, sex, median household income, smoking, and cocaine abuse to assess the odds of AMI incidence in marijuana users (Table 3). The multivariate regression models for inpatient mortality was adjusted for confounding factors such as age, race, the length of stay, the median house of income in the zip code, an indicator of sex, hospital bed size, smoking and cocaine abuse. The trends of baseline demographics, AMI prevalence and complications from 2010-2014 were measured by Cochran–Armitage test. All statistical analyses were executed by IBM SPSS Statistics 22.0 (IBM Corp, Armonk, New York). We used weighted data to produce national estimates in our study. Institutional Review Board authorization was not needed in this study because NIS database does not contain patients’ identification details.

**Table 3:** Multivariate Predictors of Acute Myocardial Infarction with known Marijuana Use MI: myocardial infarction, CAD: coronary artery disease, PCI: percutaneous coronary intervention, CABG: coronary artery bypass grafting.

Variables	Adjusted Odds Ratio^$^	95% Confidence Interval		P-value*
Age in years at admission				
11-25	Referent	Referent		
26-40	4.141	3.848	4.457	<0.001
41-55	9.099	8.463	9.782	<0.001
56-70	11.510	10.678	12.406	<0.001
Indicator of Sex				
Male vs. Female	1.507	1.465	1.549	<0.001
Race				
White	0.802	0.750	0.857	<0.001
Black	0.697	0.651	0.747	<0.001
Hispanic	0.702	0.649	0.760	<0.001
Asian or Pacific Islander	0.766	0.659	0.889	<0.001
Native American	0.693	0.600	0.802	<0.001
Other	Referent	Referent		
Comorbidities^#^				
Smoking	1.695	1.650	1.742	<0.001
Cocaine	1.106	1.073	1.140	<0.001
AIDS	1.221	1.083	1.377	0.001
Congestive Heart Failure	0.789	0.748	0.831	<0.001
Chronic Pulmonary Disease	0.838	0.814	0.862	<0.001
Coagulopathy	1.663	1.582	1.749	<0.001
Diabetes, Uncomplicated	0.941	0.911	0.971	<0.001
Diabetes with Complications	0.797	0.753	0.844	<0.001
Drug Abuse	16.182	13.824	18.942	<0.001
Hypertension	1.536	1.495	1.579	<0.001
Hypothyroidism	0.708	0.666	0.753	<0.001
Liver Disease	0.702	0.664	0.742	<0.001
Fluid and Electrolyte Disorders	1.278	1.245	1.313	<0.001
Obesity	1.264	1.222	1.307	<0.001
Peripheral Vascular Disorder	1.676	1.538	1.827	<0.001
Pulmonary Circulation Disorder	0.734	0.652	0.827	<0.001
Renal Failure	1.057	1.014	1.103	0.010
Valvular Diseases	0.692	0.629	0.761	<0.001
Dyslipidemia	3.275	3.188	3.363	<0.001
Atherosclerosis	0.849	0.773	0.931	0.001
Cardiomyopathy	2.715	2.612	2.823	<0.001
Cerebrovascular Disease	0.668	0.621	0.718	<0.001
Oral Contraceptive Pills	0.726	0.583	0.905	0.004
Thromboembolism	0.775	0.728	0.824	<0.001
Previous MI	1.101	1.056	1.148	<0.001
Family History of CAD	4.481	4.315	4.652	<0.001
Previous PCI	2.443	2.344	2.546	<0.001
Previous CABG	0.896	0.839	0.957	0.001
History of Sudden Cardiac Arrest	3.899	3.313	4.589	<0.001
*Significant P-value ≤ 0.05 at 95% Confidence Interval, ^#^Variables are AHRQ Co-morbidity measures. ^$^Regression model adjusted for age, race, sex, drug abuse, cocaine abuse, other relevant comorbidities and hospital characteristics				

## Results

Study population

We identified 2,451,933 weighted hospitalized AMI patients from the 2010 –2014 NIS database. Of these, there were 35,771 patients with a history of marijuana and 2,416,162 patients without a history of marijuana use. The mean age (years) was 49.3 ± 10.7 in the AMI-marijuana group as compared to 57.7 ± 8.9 in AMI non-marijuana group. AMI-marijuana group consisted more of younger, male (76.9% vs. 66.0%), black (33.0% vs. 13.8%) patients with more Medicaid enrollees (26.9% vs. 11.9%) compared to the non-marijuana group. In White, Hispanic and Asian ethnicities, males had a higher prevalence of AMI with marijuana use while in the Black race, females had a higher prevalence of AMI in marijuana users. The age group 41-55 years had the highest prevalence (50.1%) of AMI with marijuana use. 

The disposition by 'against medical advice' was higher in AMI with marijuana patients (4.0% vs.1.2%). Urban – teaching hospital documented more of AMI patients with marijuana use (61.7% vs. 54.4%). The low-income group ($1 - $39,999) had a higher number of AMI-marijuana group patients as compared to AMI-non marijuana group (44.0% vs. 32.2%). Weekend hospital admissions were increased in AMI with marijuana patients (28.4% vs. 25.8%) as compared to AMI without marijuana patients. Inpatient mortality rate was found lower in AMI with marijuana group (3.4% vs. 5.8%) (Table 1).  

Co-morbidities such as cardiomyopathy, previous myocardial infarction (MI), family history of coronary artery disease, chronic pulmonary disease, other neurological disorders, depression, psychosis, alcohol abuse, drug abuse, smoking, cocaine abuse, amphetamine abuse, fluid and electrolytes disorders, and liver disease were more prevalent in the AMI with marijuana group (all p<0.001) (Table 2). 

**Figure 2:**
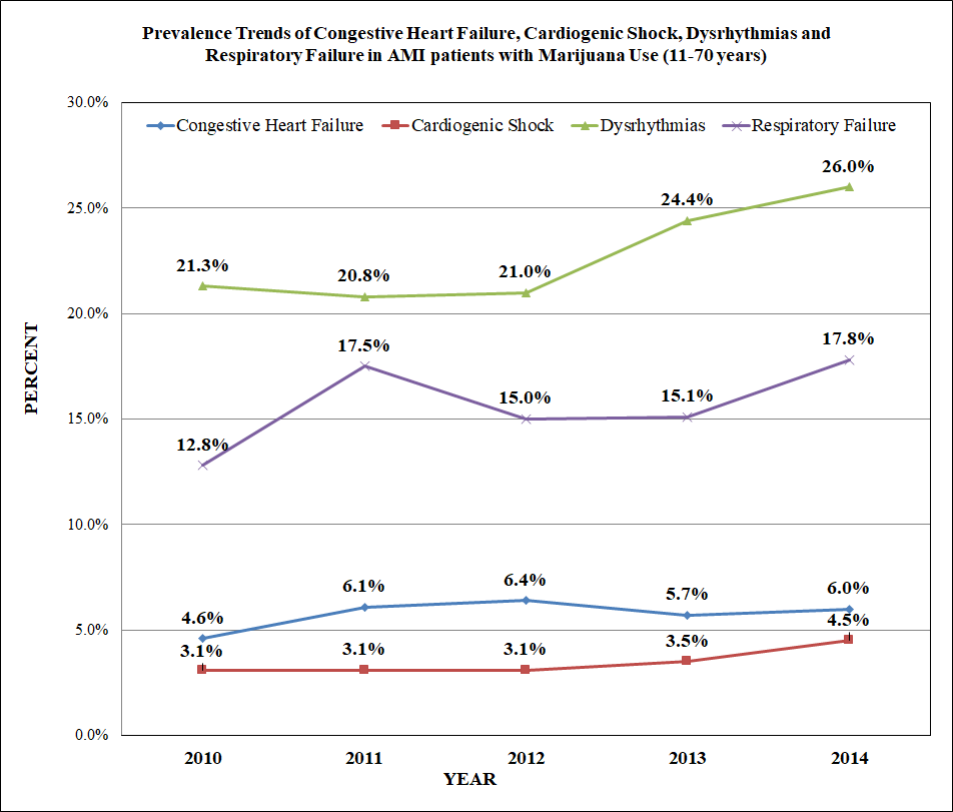
Prevalence Trends of Congestive Heart Failure, Cardiogenic Shock, Dysrhythmias and Respiratory Failure in AMI Patients with Marijuana Use (11-70 years)

The prevalence trends of dysrhythmias, respiratory failure, cardiogenic shock, and congestive heart failure increased by 4.7%, 6.4%, 1.4%, 1.4%, respectively from 2010 to 2014 (p<0.0001) (Figure 2).

Year-wise distribution of baseline demographics, comorbidities, and prevalence of complications in AMI with known marijuana use patients are shown in Table 4.

**Table 4:** Year-wise Baseline Demographics and Comorbidities in Acute Myocardial Infarction Patients with known Marijuana Use

Years	2010	2011	2012	2013	2014	P– value*
Variables	%	%	%	%	%	
Age in years at admission						<0.001
11-25	3.3	2.7	2.6	2	2.3	
26-40	19.1	18.9	18.6	17.3	16.7	
41-55	52.8	52.2	49.5	48.5	48.9	
56-70	24.7	26.2	29.3	32.1	32.2	
Indicator of Sex						<0.001
Male	81.1	78.5	76.6	75.8	74.5	
Female	18.9	21.5	23.4	24.2	25.5	
Admission Day						<0.001
Monday-Friday	70.6	74.5	73.3	69.3	71	
Saturday-Sunday	29.4	25.5	26.7	30.7	29	
Type of Admissions						<0.001
Non-elective	93.7	93.5	95.4	96.3	94.6	
Elective	6.3	6.5	4.6	3.7	5.4	
Died during hospitalization						<0.018
Did not die	96.8	96.4	96.9	96.9	96.2	
Died	3.2	3.6	3.1	3.1	3.8	
Race						<0.001
White	54.8	54.2	54	55.8	57.2	
Black	33.9	33.4	33.4	33.4	31.5	
Hispanic	7.6	7.3	6.4	5.9	6.9	
Asian or Pacific Islander	0.7	0.6	0.7	0.8	0.8	
Native American	0.9	0.7	1.4	0.6	0.7	
Other	2.1	3.8	4	3.4	2.9	
Length of stay						<0.001
≤3 days	59.8	57.5	58.1	59.3	58.9	
4 to 6 days	21.3	22.4	22.8	21.5	21.2	
7 to 9 days	7.6	9.8	9.2	8	9.2	
10 to 12 days	5.6	4.2	3.8	5	4.3	
≥13 days	5.8	6.2	6.2	6.2	6.3	
Bed Size of Hospital						<0.001
Small	8.1	7.5	8.2	9.5	14.6	
Medium	17.8	19.5	25.1	25.6	30.2	
Large	74.2	73	66.7	65	55.1	
Location/teaching status of hospital						<0.001
Rural	9.6	6.8	5.5	5.8	4.9	
Urban - non teaching	35.1	38.7	34.2	34.1	22.5	
Urban - teaching	55.3	54.5	60.2	60.1	72.6	
Region of hospital						<0.001
Northeast	14.6	16.8	16	13.3	14.4	
Midwest or North Central	26	24.3	26.5	28.1	29.2	
South	39.2	37.5	36.1	37.8	38.7	
West	20.3	21.4	21.4	20.8	17.7	
Comorbidities^#^						
Alcohol abuse	25.5	24	21.8	23.3	19.8	<0.001
Deficiency anemias	10.1	12.2	12.4	11.2	13.8	<0.001
RA/CVD	1.4	1.1	1.6	1.1	2	<0.001
Congestive heart failure	4.6	6.1	6.4	5.7	6	<0.001
Chronic pulmonary disease	20.4	20.3	22.1	23.7	24.3	<0.001
Coagulopathy	5.6	6.7	5.5	5.5	6.7	<0.001
Diabetes, uncomplicated	15.3	17	19.1	19	20	<0.001
Diabetes with chronic complications	3.1	4.4	4.7	4.7	5.7	<0.001
Hypertension	61.6	59.2	62	64.9	65	<0.001
Hypothyroidism	2.9	3	4.3	3.8	4	<0.001
Liver disease	3.3	6	4	4.5	4.6	<0.001
Fluid and electrolyte disorders	22.2	27.6	28.4	28.1	28.7	<0.001
Other neurological disorders	5.8	6.5	5.9	6.7	7	0.009
Obesity	12.6	14.5	15.4	15.8	17.1	<0.001
Peripheral vascular disorders	6.9	7.4	8.1	7.9	8.7	0.002
Psychoses	8.2	7.3	7.2	9.7	9.2	<0.001
Pulmonary circulation disorders	0.9	0.6	0.9	1.4	1	<0.001
Renal failure	7.3	9.7	11.4	9.4	12.4	<0.001
Valvular disease	1.4	1.5	1.7	1.2	2	0.001
Weight loss	3.1	3.2	3.9	3.8	4	0.01
Dyslipidemia	49.1	49.4	51.1	52.6	50.1	<0.001
Atherosclerosis	5.2	6.4	6.8	6.6	7.4	<0.001
Cardiomyopathy	11.9	11.6	13.1	11.6	12.3	0.043
Cerebrovascular disease	3.1	3.1	2.2	2.5	2.5	0.006
Thromboembolism	3	3.9	3.2	3.2	4	0.001
Previous MI	13.1	10.5	12.2	14.2	13.9	<0.001
Family history of CAD	12	13.5	13.6	14.2	14.3	0.001
Previous PCI	13.7	12.8	14.6	14.8	15.8	<0.001
History of SCA	0.5	0.4	0.7	0.5	1.2	<0.001
Cardiogenic shock	3.1	3.1	3.1	3.5	4.5	<0.001
Smoking	76.1	74.6	75.3	76.4	76.9	0.011
Cocaine	22.1	21.3	18.3	17.5	16.8	<0.001
Amphetamine	4.6	5.3	6.4	5.5	6	<0.001
Dysrhythmia	21.3	20.8	21	24.4	26	<0.001
Respiratory Failure	12.8	17.5	15	15.1	17.8	<0.001
Pneumonia	6.1	7.6	5.8	7.6	7.4	<0.001
Septicemia	3.1	4.8	4.5	5.1	6.5	<0.001
Hemodialysis	2.3	3	2.4	2.6	3.3	0.001
Obstructive Sleep Apnea	3.8	4.1	5.4	5.3	6.6	<0.001
Vasopressor infusion	1.4	0.7	1	0.9	1.4	<0.001
*Significant P-values ≤ 0.05 at 95 confidence Interval, ^#^Variables are AHRQ Co-morbidity measures						
Abbreviations: AIDS – Acquired Immunodeficiency Syndrome, RA/CVD – Rheumatoid Arthritis/ Collagen Vascular Disease, MI – Myocardial Infarction, CAD – Coronary Artery Disease, PCI – Percutaneous Coronary Intervention, CABG – Coronary Artery Bypass Grafting, SCA – Sudden Cardiac Arrest.						

Odds of AMI incidence in marijuana use

The odds of developing AMI in marijuana users were considerably higher in the male sex (OR=1.507, CI 1.465 - 1.549, p<0.001) and the 56 – 70 years’ age group (OR 11.510, CI 10.678 - 12.406, p<0.001). Drug abuse (OR 16.182, CI 13.824 - 18.942, p<0.001), family history of CAD (OR 4.481, CI 4.315 - 4.652, p<0.001), dyslipidemia (OR 3.275, CI 3.188 - 3.363, p<0.001), cardiomyopathy (OR 2.715, CI 2.612 - 2.823 , p<0.001), previous PCI (OR 2.443, CI 2.344-2.546, p<0.001), smoking (OR 1.695, CI 1.650-1.742, p<0.001), peripheral vascular disorder (OR 1.676, CI 1.538-1.827, p<0.001), and coagulopathy (OR 1.663, CI 1.582-1.749, p <0.001) increased the odds of developing AMI in marijuana users (Table 3). Multivariable analysis showed that marijuana use was a significant risk factor for AMI development when adjusted for age, sex, race (adjusted OR 1.079, 95% CI 1.065-1.093, p<0.001); adjusted for age, female, race, smoking, cocaine abuse (adjusted OR 1.041, 95% CI 1.027-1.054, p<0.001); and also when adjusted for age, female, race, payer status, smoking, cocaine abuse, amphetamine abuse and alcohol abuse (adjusted OR: 1.031, 95% CI: 1.018-1.045, p<0.001). Thus, overall marijuana use was associated with a 3-8% increased the risk of AMI.

Length of stay & total hospital charges

The mean length of stay (days) (4.7±5.9) vs. 5.6±8.0)) and total hospital charges ($76,272.23 vs. $85,702.22) were lower in AMI with marijuana group. The total mean hospital charges were increased from 2010 to 2014 whereas, there was no major change in the mean length of stay from 2010 to 2014. The mean length of stay was almost equivalent in females and males ((4.8±5.7) vs. (4.7±6.0)).  All p-values were <0.001.

In-hospital mortality

**Figure 3:**
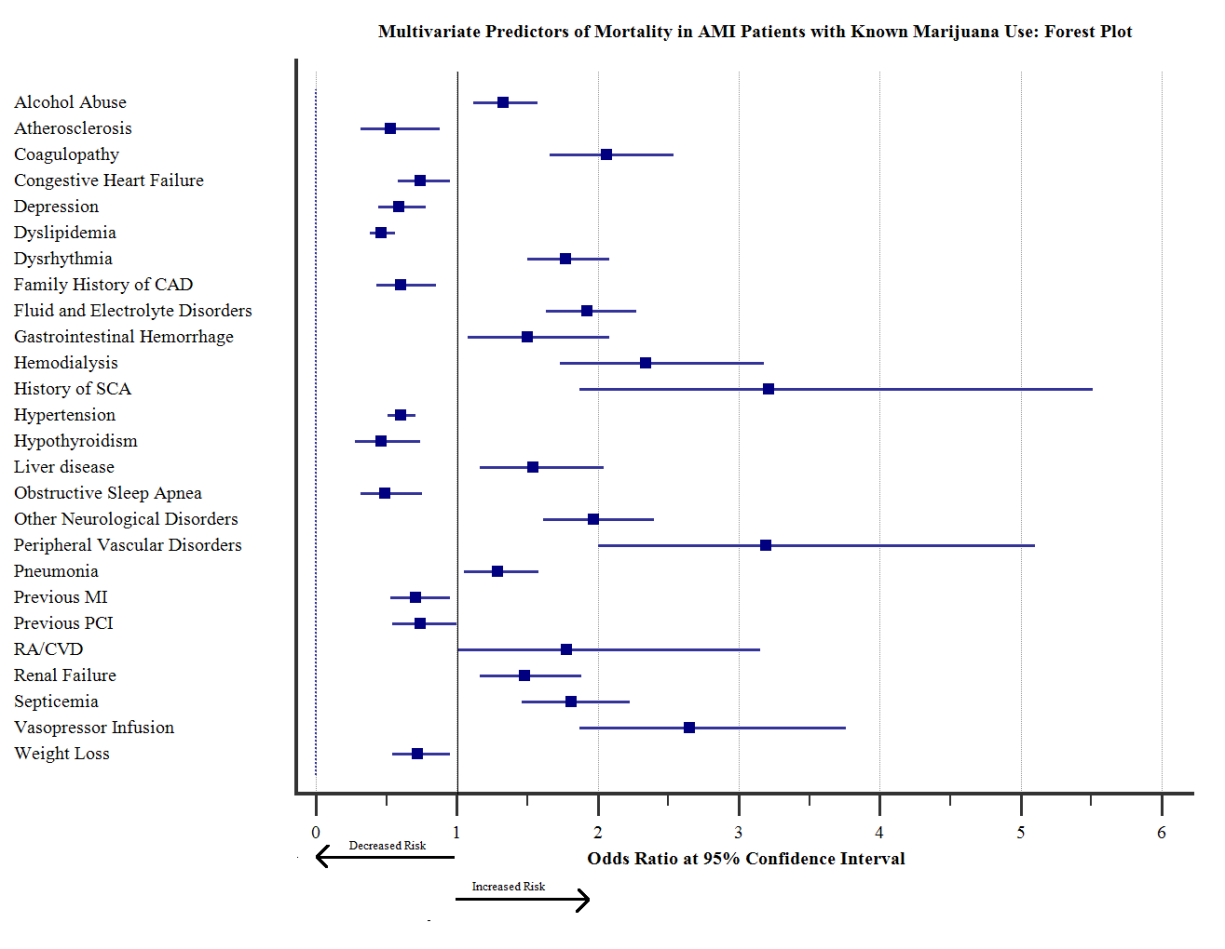
Multivariate Predictors of Mortality in AMI Patients with known Marijuana Use: Forest plot The top independent predictors of mortality were respiratory failure (OR 18.895, CI 15.572 – 22.928, p<0.001), cerebrovascular disease (OR 8.977, CI 6.905-11.671, p<0.001), metastatic cancer (OR 7.055, CI 3.482 - 14.294, p<0.001), cardiogenic shock (OR 6.008, CI 4.888 – 7.385, p<0.001), solid tumor without metastasis (OR 3.673, CI 2.054 - 6.570, p<0.001), vasopressor infusion (OR 2.649, CI 1.866-3.761, p<0.001), and hemodialysis (OR 2.342 CI 1.725–3.179, p<0.001). The other predictors are shown in forest plot.

Among all age groups, the 41-55 years’ group showed the highest mortality (46.3%) related to AMI in marijuana users. The mortality in females was found to be higher in White and Black races, whereas Asian and Native American ethnicities showed male predominance. The trend of inpatient mortality pertinent to AMI in marijuana users was significantly increased by 14.7% from 2010 to 2014 (trend p<0.0001) (Figure 1), however; the odds of mortality was not significantly increased in AMI-marijuana group as compared to AMI- non-marijuana group (adjusted OR 0.742, CI 0.693-0.795, p<0.001). The top independent predictors of mortality were respiratory failure (OR 18.895, CI 15.572 – 22.928, p<0.001), cerebrovascular disease (OR 8.977, CI 6.905-11.671, p<0.001), metastatic cancer (OR 7.055, CI 3.482 - 14.294, p<0.001), cardiogenic shock (OR 6.008, CI 4.888 – 7.385, p<0.001), solid tumor without metastasis (OR 3.673, CI 2.054 - 6.570, p<0.001), vasopressor infusion (OR 2.649, CI 1.866-3.761, p<0.001), and hemodialysis (OR 2.342 CI 1.725–3.179, p<0.001). The other predictors are shown in Figure 3.

## Discussion

In this retrospective analysis, after adjusting the potential confounding factors in different regression models, we reported that use of marijuana linked to amplify the risk of AMI 3- 8% irrespective of the duration of marijuana consumption. The previous study has also shown that risk of AMI is amplified in the individuals who smoke marijuana [4]. There were 13 cases of chest pain and MI which were associated with marijuana smoking [9]. However, the previous studies had the small sample size and did not take the confounding factors into account in the results. Our study utilized the largest inpatient database of the United States which can measure the more accurate risk of AMI in marijuana users. In our study, marijuana use in AMI did not increase overall mortality. Current literature also supports that individuals with long-term use of marijuana may have no increased risk of mortality in the age group of younger than 50 years [10]. According to The Substance Abuse and Mental Health Statistics National Survey [11] for 2006, the substance abuse dependence among adolescents older than 12 years of age and adults was twice more in males than females (12.3% vs. 6.3%). The epidemiology of substance abuse has changed in past few decades, with an increase in girls’ substance abuse [12]. Our study revealed steadily increasing trends (year-wise frequency) of AMI incidence with marijuana use in the female group from 2010 to 2014.

Our study showed that AMI with marijuana group consisted more of younger, male (76.9% vs. 66.0%), African American (33.0% vs. 13.8%) patients compared to the non-marijuana group. The same result has been documented previously [2, 9]. The higher weekend admissions in AMI-marijuana users could be related to recreational substance use more during weekends leading to higher AMI during the same time. The shorter length of stay (LOS) in days and lesser hospital charges in the AMI-marijuana group can be explained due to higher patient disposition against medical advice as compared to the AMI-non marijuana group. In our study, Medicaid is the primary payer in the majority of AMI-marijuana group patients which could be explained by a higher number of low-income patients affected by AMI and forced to have costly substance abuse treatment through Medicaid [8]. The main biological effects of smoking marijuana are mediated by the cannabinoids (CBs) receptors (CB1, CB2) which are located in the central nervous system, heart, adrenal gland, lung, liver [13].   

Our study revealed that previous history of MI was documented higher in AMI with marijuana group. In patients with past medical history of coronary artery disease, marijuana appears to produce angina symptoms sooner after exertion as compared to tobacco smoking [14]. The family history CAD which is originally risk factor for AMI was also found to be higher in hospitalized AMI-marijuana group patients. As the previous study has also described depression, psychosis, alcohol abuse, smoking, cocaine abuse, amphetamine abuse and drug abuse were found to be higher with marijuana as compared to non-marijuana users [15].  In addition to the impact of cocaine abuse on stroke [16], an association between cannabinoids use and neurovascular complications have also been reported [17]. In an animal experiment done on rats, THC has shown toxic effects on the mitochondria of the brain by the generation of hydrogen peroxide and reactive oxygen radicals, which is one of the mechanisms involved in stroke in humans [18, 19]. Our study showed increasing year-wise-frequency of dysrhythmias, respiratory failure, cardiogenic shock, and congestive heart failure in AMI patients with marijuana use between 2010 to 2014 which could be because of inhaled marijuana generating more carboxyhemoglobin that could interfere in cellular oxygenation [20]. Poor oxygenation to myocardium and lungs could be the explanation for increased cardiovascular diseases with marijuana use [21]. The dysrhythmias after marijuana smoking can be due to increased sympathetic and decreased parasympathetic activity by tetrahydrocannabinol (THC) [22]. The tachycardia is the most consistent finding after smoking marijuana because of increased sinus node automaticity [23]. Beta-adrenergic stimulation by marijuana induces sinus tachycardia in humans [24]. Cannabis usage can lead to ischemic episodes and tachyarrhythmias [22, 25-27], it can also result in an increase in parasympathetic tone and parasympathetic activity which cause sudden asystolic arrest causing death [28]. The obstructive lung diseases could result from long-time exposure to marijuana smoking [29]. Chronic obstructive pulmonary disease (COPD) is more common among AMI patients (13.2%) with extended LOS, higher mortality, and more adverse outcomes as compared to those without COPD [30].

This study has a few limitations. Over-reporting or under-reporting the estimated population is possible in this study because of ICD-9 coding errors in an administrative database such as NIS. The time for which patients used marijuana was not specified in the database so we could only measure lifetime risk of AMI in marijuana users. We cannot follow up our study population due to nature of the data. The limitations can be overlooked if the benefits of the large database are weighed against it to analyze marijuana impact on cardiovascular disease.

## Conclusions

In our study, the lifetime AMI odds were increased up to 8% in recreational marijuana users. Overall odds of mortality were not increased significantly in AMI with marijuana group. However, marijuana users showed higher trends of AMI prevalence and AMI related mortality from 2010 to 2014. It is crucial to assess marijuana-related cardiovascular effects before we can fully explore therapeutic use. We aim to demonstrate the importance of patient history, including recreational drug use, in identifying the etiology of an otherwise unexplained myocardial infarction. The important role of clinicians in educating patients about the potential risks involved in marijuana overuse needs to be emphasized amidst legalization of marijuana use for therapeutic purposes.

## Appendices

**Table 5:** Appendix: ICD-9-CM and CCS Codes Used to Identify Comorbidities, In-Hospital Procedures, and Complications

Risk Factors/ Co-morbidity	Source	Codes
Smoking	ICD-9	V15.82, 305.1
Dyslipidemia	CCS	53
Family history of CAD	ICD-9	V17.3
Prior myocardial infarction	ICD-9	412
Prior PCI	ICD-9	V45.82
Prior CABG	ICD-9	V45.81
Atrial fibrillation	ICD-9	427.31
Acute respiratory failure	ICD-9	518.81, 518.82, 518.84, 799.1, 786.09, 518.4, 518.5
Cardiogenic shock	ICD-9	785.51
Atherosclerosis	CCS	114
Congestive heart failure	CCS	108
Cardiomyopathy	CCS	97
History of Sudden Cardiac Arrest	ICD-9	V12.53
Cerebrovascular disease	CCS	109
Family History of Stroke	ICD-9	V17.1
Transient ischemic attack	CCS	112
Oral contraceptive use	CCS	176
Thromboembolism	CCS	118
Hemodialysis	CCS	58
Motor Vehicle Accident	ICD-9	E810, E811, E812, E813, E814, E815, E816, E817, E818, E819
Septicemia	CCS	2
Dressler’s Syndrome	ICD-9	411.0
Left Ventricular Failure	ICD-9	428.1
Vasopressor Infusion	ICD-9	00.17
Obstructive Sleep Apnea	ICD-9	327.23, 780.50, 780.51, 780.53, 780.57, 780.59, or 786.03
Pneumonia	CCS	122
Cocaine	ICD-9	304.20–304.22; 305.60–305.62
Amphetamine	ICD-9	304.40–304.42; 305.70–305.72
Alcohol Abuse	ICD-9	303.00–303.02; 303.90–303.92; 305.00–305.02
Abbreviations: ICD‐9‐CM= International Classification of Diseases, Ninth Revision, Clinical Modification; CCS= Clinical Classification Software.		
